# Diagnosis of deep vein thrombosis using 3D black-blood thrombus imaging (BTI): preliminary clinical experience

**DOI:** 10.1186/1532-429X-18-S1-Q58

**Published:** 2016-01-27

**Authors:** Hanwei Chen, Guoxi Xie, Jianke Liang, Wei Deng, Zhuonan He, Yufeng Ye, Xueping He, Qi Yang, Xiaoming Bi, Xin Liu, Debiao Li, Zhaoyang Fan

**Affiliations:** 1 Department of Radiology, Guangzhou Panyu Central Hospital, Guangzhou, China; 2 grid.458489.c0000000104837922Shenzhen Institutes of Advanced Technology, Shenzhen, China; 3 grid.413259.80000000406323337Xuanwu Hospital, Beijing, China; 4 grid.50956.3f0000000121529905Biomedical Imaging Research Institute, Cedars-Sinai Medical Center, Los Angeles, CA; 5 Siemens Healthcare, Los Angeles, CA USA

## Background

Deep vein thrombosis (DVT) is a common but elusive illness that can lead to fatal pulmonary embolism and sudden death. Effective treatment of DVT requires accurate evaluation of thrombus distribution and stage. MRI is one of diagnostic imaging modalities for DVT, and two conventional methods are MPRAGE[1] and CE-MRV[2]. Recently, 3D T1-weighted variable-flip-angle turbo spin-echo (SPACE) was proposed as a black-blood technique that permits more direct visualization of DVT[3]. However, signal suppression of tremendously slow venous blood flow remains a challenge for SPACE. The unsuppressed blood signal could be a confounder in thrombus detection[3]. We hypothesized that the 3D black-blood thrombus imaging (BTI) technique[4] that combines SPACE with DANTE black-blood preparation[5] (DANTE-SPACE) might address the above issue.

## Methods

### Experiment

The IRB-approved study was performed on a 3T scanner (Siemens TimTrio, Germany). DANTE-SPACE was first optimized on 8 healthy subjects (4 F 4 M, age 25 ± 4) and then tested on 12 patients (6 F 6 M, age 52 ± 13) with DVT. The optimized parameters for DANTE included: FA 15°, pulse trains 175, RF gap 1 ms, gradient 20 mT/m. The parameters for SPACE included: 3D coronal imaging with a resolution of 1.1 × 1.1 × (1.1-1.3)mm^3^ (interpolated to 0.55 × 0.55 × [0.55-0.65] mm^3^),TR/TE 650/9.8 ms, turbo factor 40, GRAPPA 2, scan time ~4 min. The scan was targeted to the thrombus region that was pre-determined by ultrasound within 3 days. Conventional SPACE, MPRAGE, and CE-MRV were conducted for comparison.

### Image Analysis

two radiologists (J. L. and Y. Y.) evaluated randomized images and gave the diagnosis confidence scores (1-poor, 4-excellent) to each technique independently. The sensitivity (SE), specificity (SP), positive and negative predictive values (PPV and NPV), and the accuracy (ACC) of DANTE-SPACE, SPACE, and MPRAGE were calculated using CE-MRV as the reference. The diagnostic agreement between DANTE-SPACE/SPACE/MPRAGE and CE-MRV and the interobserver agreement were conducted using Cohen κ test.

## Results

**Figure 1:**
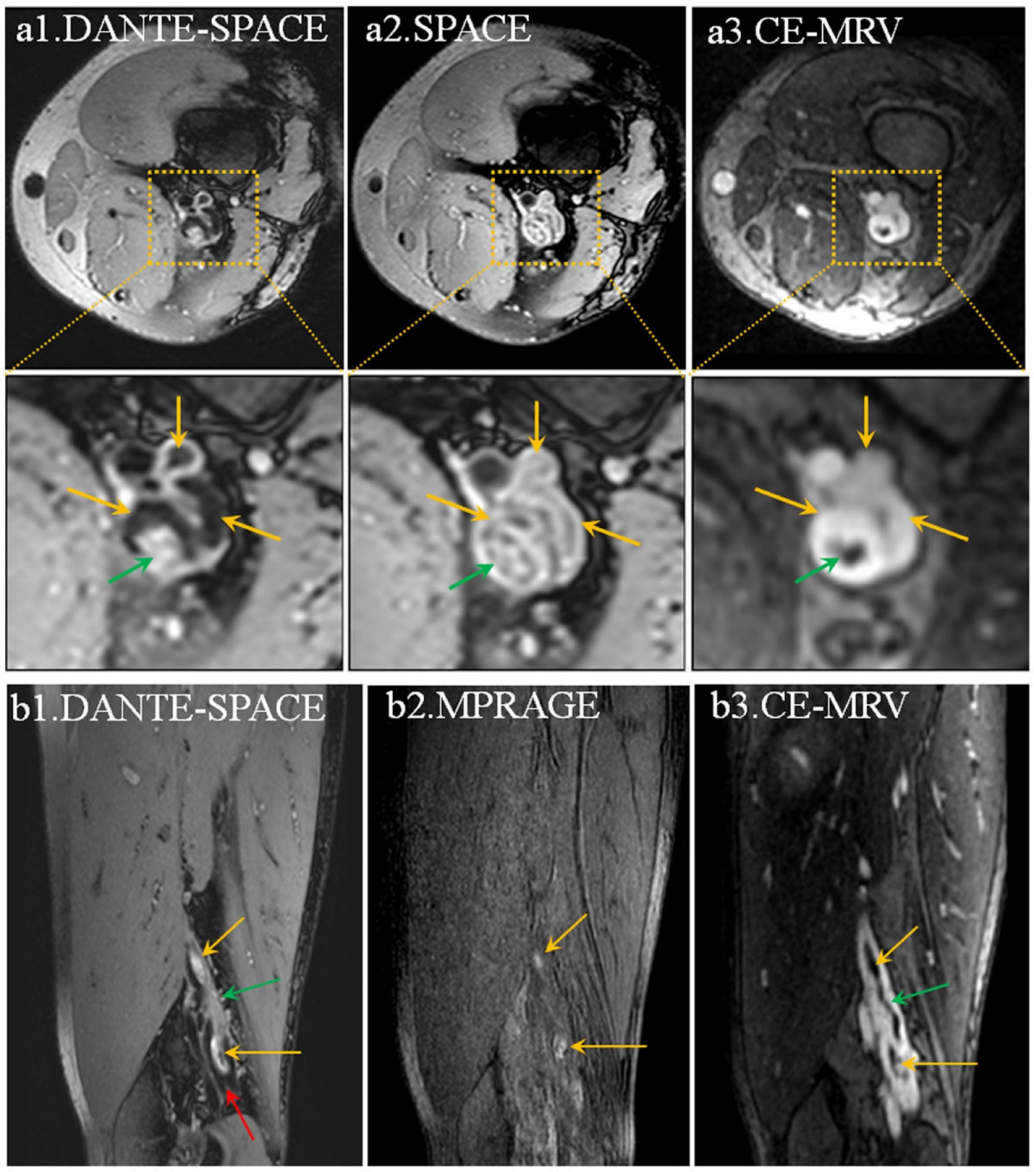
**Representative images from a patient subject**. The thrombus-mimicking venous blood signal with the SPACE sequence can be effectively eliminated by DANTE-SPACE (yellow arrows on a1&a2). MPRAGE only detected the DVT in the acute or sub-acute stage because of short T1 relaxation time (yellow arrows on b2), while DANTE-SPACE depicted the DVT well regardless of the thrombus stage (yellow and green arrows on b1) as the venous blood flow (red arrow on b1) around the thrombus was effectively suppressed. The thrombus distribution matched well between DANTE-SPACE and CE-MRV (a1 vs. a3, b1 vs. b3).

Compared to SPACE, DANTE-SPACE effectively nulled the residual blood that would otherwise be mistaken as part of thrombus (Fig. 1a1&a2). In contrast to MPRAGE that is only sensitive to the acute or sub-acute thrombus (Fig. 1b1&b2), DANTE-SPACE was able to depict the DVT regardless of the thrombus stage. DANTE-SPACE provided the highest diagnosis confidence score, when compared to SPACE and MPRAGE, and high SE, SP, PPV, NPV and ACC (Table 1).

**Table 1:** Qualitative and quantitative analysis results for the comparison among DANTE-SPACE, SPACE, MPRAGE, and CE-MRV

	Score (mean ± std)	Number of thrombosed segment	SE (%)	SP (%)	PPV (%)	NPV (%)	ACC (%)	Diagnostic agreement (κ value / p)	Interobserver agreement (κ value / p)
DANTE-SPACE (reader1)/(reader2)	(3.60 ± 0.61)/(3.70 ± 0.46)	22 / 19	90.9 / 94.4	97.8 / 97.9	90.9 / 89.5	97.8 / 98.9	96.4 / 97.3	(0.89 / < 0.01) / (0.90 / < 0.01)	0.73 / <0 .01
SPACE (reader1)/(reader2)	(3.22 ± 0.88)/ (2.72 ± 1.02)	20 / 20	86.4 / 83.3	94.4 / 98.9	78.3 / 92.9	95.5 / 94.9	92.0 / 94.6	(0.88 /< 0.01) / (0.75 / < 0.01)	0.63 / <0.01
MPRAGE (reader1)/(reader2)	(1.94 ± 0.79)/ (2.61 ± 0.90)	23 / 14	81.8 / 72.2	98.8 / 94.7	95.0 / 75.0	96.7 / 96.7	96.4 / 92.9	(0.75 / < 0.01) / (0.78 / < 0.01)	0.71 / <0.01
CE-MRV (reader1)/(reader2)	(3.81 ± 0.42)/ (3.82 ± 0.41)	22 / 18	----	----	----	----	----	----	0.81 / <0.01

## Conclusions

DANTE-SPACE is a BTI technique providing excellent venous blood signal suppression and definitive thrombus detection. The preliminary patient study has demonstrated that the technique may outperform SPACE, MPRAGE and potentially become a noncontrast alternative to CE-MRV in the diagnosis of DVT.

## References

[1] Moody (1998). Radiol.

[2] Arnoldussen C (2014). Phlebology.

[3] Treitl (2015). Invest Radiol.

[4] Fan (2015). ISMRM.

[5] Li L (2012). MRM.

